# A novel RFID multi-tag anti-collision protocol for dynamic vehicle identification

**DOI:** 10.1371/journal.pone.0219344

**Published:** 2019-07-05

**Authors:** Zhijian Qu, Xubing Sun, Xinqiang Chen, Shengao Yuan

**Affiliations:** 1 Electrical and Automation Engineering College, East China Jiaotong University, Nanchang, Jiangxi, China; 2 Institute of Logistics Science and Engineering, Shanghai Maritime University, Shanghai, China; Central South University, CHINA

## Abstract

In order to obtain the information of the vehicle tags in adverse traffic conditions, we proposed a novel reservation framework named reservation to cancel idle-dynamic frame slotted ALOHA (RTCI-DFSA) algorithm. Firstly, the framework employed reservation mechanism to remove idle slot, and thus improve the system identification efficiency. Secondly, the vehicle information was identified by the tag serialization polling identification method. The experimental results showed that the proposed RTCI-DFSA algorithm performed better than the traditional frame slotted ALOHA (FSA) and dynamic frame slotted ALOHA (DFSA) algorithms. More specifically, the tag loss rate of the proposed framework is significantly lower than the frame length fixed and conventional dynamic vehicle identification algorithms. In addition, the experimental results demonstrated that the throughput rate of the proposed algorithm increased from 0.368 to 0.6. Besides, the identification efficiency and applicability of the proposed framework were both higher than other tag identification algorithms.

## Introduction

In recent years, the rapid automobile expansions and severe traffic congestions motivated development of intelligent transport system (ITS). With the advantages of convenience, rapidity and reliability (which is mainly composed by the intelligent vehicle identification system (IVIS)), ITS is widely used in many situations such as urban traffic control, parking lot management, congestion warning and automobile anti-theft system. The existing IVIS employed computer vision to detect vehicles, which may obtain many false alarms in adverse weather [1–3]. Specifically, interferences from the external environment (especially in foggy days) can impose strong negative effects on the system, leading to traffic monitoring incidents [4]. Therefore, solving the problem of intelligent identification of vehicle information in severe weather conditions or in complicated traffic states have become a hot research topic in the community.

Based on the sensor detection mechanisms, the vehicle identification system used along the expressways can be divided into the image-based and the radio frequency identification (RFID) electromagnetic induction-based vehicle identification system [5]. The image-based identification system shoot pictures of the passing vehicles plates via cameras installed on both sides of the portal platoons along the expressways, and then use the computer vision technology to identify the numbers in the plates. In fact, the identification method can accurately capture the plate information. Nevertheless, computer vision based techniques can hardly obtain satisfied performance in hazy weather, as images are with low contrast and blurred colors due to severe degeneration, and a few distinct features can be extracted [6, 7]. Therefore, traditional computer vision methods cannot meet the requirements on the vehicle information identification under severe weather conditions.

Compared with other electronic sensing technologies, the RFID electromagnetic induction-based vehicle identification technology has the advantages of low cost, long durability, strong anti-interference capability and high resistance to the external environment, and thus is widely deployed in various scenarios. The coupling modes of the previous RFID systems can be divided into categories of the inductive coupling and the electromagnetic backscattering modes. The typical range of the inductive coupling mode is less than 1cm in a closed coupled system. In the remote coupling system, the maximum RFID controlling distance between the reader and the tag is 100 cm. Therefore, the inductive coupling mode is usually deployed in scenarios with short operating distance. The electromagnetic backscattering mode transmits the radio frequency energy through a radar model, with maximum operating distance reaches 20 m. It is usually set in vehicle identification situations requires long identification distance. According to references [4, 5, 8], the existing vehicle identification technology is mainly laid out in the intelligent toll collection systems, and its potential application in the intelligent identification systems used along the expressways has not been studied yet. The main reason is that vehicles move fast on the expressways, and when several vehicles parallel pass through the reader identification area, the information on the tags are uploaded to the reader through a shared wireless channel, and thus the information collision of these tags leads to the failure of vehicle information transmission. When the reader starts to detect the information in the coming round, some vehicles have moved out of the identification scope of the reader, and the vehicles information are lost, resulting in data missing outliers to the traffic management center. It is not easy to identify vehicle fast and accurately (anti-collision) by employing RFID-based automatic vehicle identification system, which can further hinder the development of the intelligent vehicle identification system.

The anti-collision algorithm of traditional time division multiple access (TDMA) multi-tag RFID system can be divided as the binary tree-based algorithm and the ALOHA (Aloha-based) algorithm [9–11]. The first type is a deterministic algorithm which was used to segment tag sets by random binary tree at the beginning stage of vehicle detection. Due to the complicated calculation procedure, the binary tree-based methods caused the reader takes a long time to give a query command, and thus sharply increased the power consumption of the reader. For example, the enhanced query tree (EQT) algorithm proposed in [12] can dynamically adjust the length of prefix code, and avoid the query of idle segments and improve the binary tree-based algorithm. The collision tree (CT) algorithm proposed in [13] adopted the Manchester coding to identify the collision bits of tag signals. Similarly, the collision tracking tree algorithm (CTTA) proposed in [14] adopted the Manchester coding-based bit tracking technology, and can detect the collision bits during the time slots of the collision. Though it improved the EQT and CT algorithms, collision still occurred from the beginning of the identification process. The CTTA algorithm inevitably increases the polling cycle and reduces the efficiency of the identification system, and thus it may fail to meet the fast identification requirements.

The second type is a stochastic algorithm based on ALOHA method. Due to the instable working interval of ALOHA, the tag randomly selects the communication slot after the reader broadcasts the query command. The theoretical throughput rate is 1/e, which is available in the reference [15]. Though the traditional ALOHA method is very simple in the calculation procedure, it has the problem of tag starvation (i.e., continuous tag collisions in more than one polling cycles). To solve the problem, many researchers have proposed varied studies to improve the traditional ALOHA. The FSA algorithm selected frame slots to enable the communication with the tags, thus solving the tag starvation problem of the ALOHA-based algorithm, more detailed can be found in [16, 17]. However, we need to predefine the relationship between the number of total tags and the frame length. The system throughput will be very low when the number of tags and frame length is uncertain. The DFSA algorithm (an improvement version of FSA [18–21]) solved the problem by the following two categories [22]. The first category is the tag number estimation algorithm, and a backlog estimation algorithm with satisfied estimation accuracy in [23, 24]. A minimum distance estimation method was proposed in [25] with estimation accuracy is up to 8% when the number of total tags is less than 80. The second category is the optimal frame length adjustment algorithm, and a two-factor dynamic frame length adjustment algorithm was proposed in [26], and in [27] the optimal frame length determination algorithm was implemented based on different slot execution time. These algorithms are designed to ensure the maximum throughput of the system and are applicable to the static tag identification models. It is recognized that the vehicle identification on the expressways are very low as the RFID reader scan the tags in static models with low efficiency [9, 28, 29]. Therefore, the existing tag recognition algorithms can hardly meet the requirements of the intelligent vehicle recognition on the expressway.

A possible solution to realize the rapid vehicle identification is to improve the identification efficiency of the system. In this study, we have carefully studied the factors deteriorating the tag identification efficiency, and proposed a novel algorithm (the RTCI-DFSA algorithm) for the vehicle identification on the expressway. In the proposed RTCI-DFSA algorithm, we developed a reservation to cancel idle slot reservation mechanism for accelerating the communication between the tags and the reader. More specifically, the reader broadcasts a request command to the tag, and thus a random number in the frame length range of the reader is generated by the self-contained counter of the tag. Based on that, the reader finish the slot reservation procedure, and then remove the slots that are not responded by tags, adjusting the efficient frame length of the system to remove all idle slots. After that, the reader and the tag communicated at the collision and the successful slot, and thus improved the system identification efficiency. In order to increase the number of polled vehicle tags and reduce the rate of tag loss [30], the system integrates tag-serialization identification technology and adopts “first come, first out” identification mechanism to ensure that vehicle information is completely identified by the reader [31–33]. Through the analysis of the above RFID anti-collision protocol, it can effectively promote the accuracy of the system for vehicle identification, reduce the loss rate of vehicle tags, and improve the practicability in harsh environments [34–37]. Thereby ensuring the completeness of the data of the traffic control department, and promoting the accuracy of road planning and decision making, thus effectively reducing the occurrence of traffic accidents. The main contributions are summarized as follows: (1) we analyzed the existing highway vehicle identification system and proposed to use RFID to identify vehicles, which improved the traditional vehicle identification system to obtain vehicle information in bad weather. (2) we proposed a new efficient tag identification algorithm, the RTCI-DFSA, applied the algorithm to rapidly identify vehicles on expressways, and effectively improve the practicality of the intelligent vehicle identification system and ensure the stability of the system. (3) we built a rapid vehicle identification model and applied the dynamic tag-serialization identification method to reduce the rate of vehicle tag loss, improve the reliability of the intelligent vehicle identification system.

## Intelligent vehicle identification system for expressways

### Framework of the intelligent vehicle identification system

**Fig 1:**
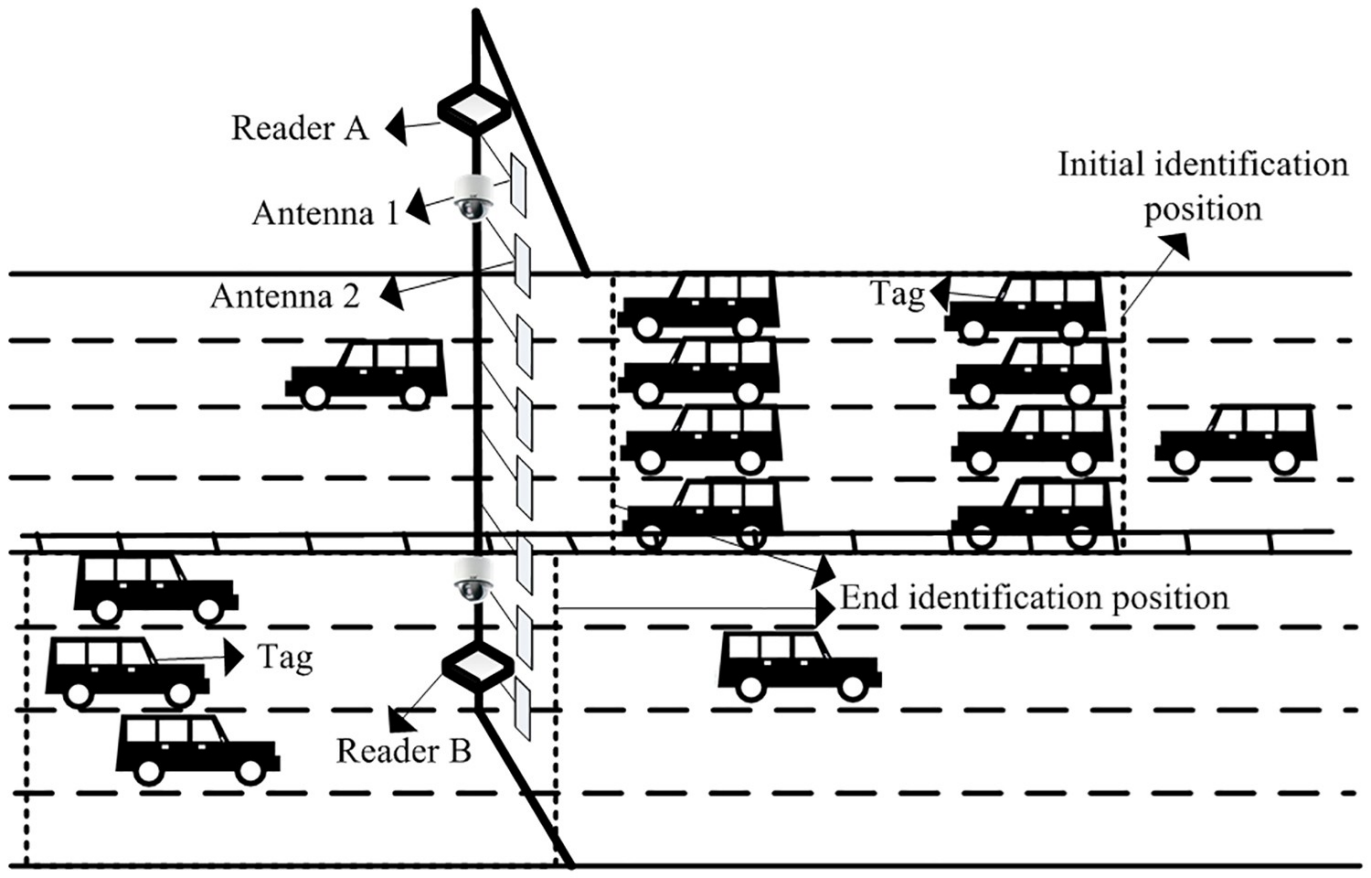
RFID intelligent vehicle identification system.

An advanced IVIS is the key subsystem of ITS. It mainly contains of antennas and readers which are deployed along expressway shoulders, and tags are placed under the vehicle windshield and a remote server. The structure of the system is shown in Fig 1.

The IVIS mainly stores and manages the vehicle tag information identified by the RFID reader through the host of the remote server, and meanwhile it remotely controls the working state of each reader. The reader is connected to the server host through RJ-45 network lines, and adjustable output power modules are installed on both sides of the dual-loop lines to prevent loss of vehicle information when electromagnetic waves are not transmitted for the expected transmission distance in bad weather. The detailed structure is shown as Reader A and Reader B in Fig 1. Among others, a single reader is connected with an external antenna through a coaxial cable, to obtain a wider radiation perspective and enhance the intensity of the reader’s broadcasting signal. The identification distance of the antenna and the speed at which the vehicle is running determine the number of vehicles within the reader’s identification range.

### Protocol of intelligent RFID-based vehicle identification system

The selection of the communication protocol between RFID tags and the readers determine the efficiency of the IVIS. This paper analyzes several existing RFID air interface protocols that is in line with the applicable international standard. It is concluded that, electronic product code (EPC) Global and international standardization organization/international electrotechnical commission (ISO/IEC) standards are used more widely the others [38]. The EPC system is a system containing EAN/UCC coding and is also an important component of the EPC, which is characterized by high communication efficiency. ISO and IEC are the main institutions for customizing global international standards. Different from the EPC Global which focuses on 860-960MHz band, ISO/IEC issued standards for each band, with different standards containing different frequencies and identification algorithms for the radio frequency identification equipment. ISO /IEC 18000-6C is a global communication standard with higher throughput for identifying tags than the ISO/IEC 18000-6B standard, while ISO/IEC 18000-6B and ISO/IEC 18000-6A standards are suitable for refinement. According to reference [39], as shown in Table 1.

**Table 1:** Anti-collision algorithms and their performance under different international standards.

Applicable frequency bands	Anti-collision algorithms	International Standard of RFID	Throughput	Complexity
HF	QT/PA/FSA	ISO/IEC 18000–3 Mode1	Low	Low
	DBSA	ISO 14443-3A	High	High
	SA	ISO/IEC 18000–3 Mode2	Low	Low
	DFSA	ISO 14443-3B	High	Medium
UHF	TS	ISO/IEC 18000-6B EPC global Class 0 EPC global Class1	High	High
	Q, FSA, DFSA	ISO/IEC 18000-6C EPC global C1G2	High	Medium
	BFSA-muting-early-end	ISO/IEC 18000-6A	Medium	High

The performance of the system is measured by the rate of successful vehicle identification. We compares and analyzes several standard protocols, and selects the basic protocol suitable for the vehicle identification by taking into account the characteristics of the IVIS for the expressway, combined with the anti-collision algorithm corresponding to the existing air interface protocols. For example, a group of tags are installed on a single vehicle, and this group of tags includes at least one tag. The identification probability of the vehicle can be expressed as follows:

$$P\{S_{Vehile}\}=1-\prod_{\forall t\in f_{Vehile}}\left(1-P\{S_{t})\right\}$$ (1)

Where: P{*S*_*Vehile*_} represents the identification probability of the vehicle, Vehile *P*{*S*_*t*_} means the identification probability of tags in a single vehicle, and *f*_*Vehile*_ refers to a group of tags in a vehicle. Among others, the identification probability of tags *P*{*S*_*t*_} depends on the number of polling cycles within the identification range of the reader, the information interaction rate with the reader and the number of tags in the tag group. Therefore, the identification probability of the tag can be expressed as Eq (2):

$$P\{S_{t}\}=1-{\left(1-(1-P\{S_{c}\})P\{S_{r}\}\right)}^{N_{r}}$$ (2)

In Eq (2),*P*{*S*_*c*_} denotes the probability of tag collision, *P*{*S*_*r*_} means the probability of successful tag identification, and *N*_*r*_ is the amount of tags identified in the identification range of the reader. Eqs (1) and (2) show that the identification performance is mainly determined by the identification efficiency and the identification accuracy of the vehicle identification system. Therefore, the protocol proposed in this paper chooses the DFSA algorithm under EPC Global C1G2 standard to improve the identification performance. The DFSA algorithm with the function of dynamic frame length adjustment, can ensure the efficiency of the identification system and facilitate system improvement and optimization.

## Reservation to cancel Idle to improve the DFSA algorithm

### Ideas of EPC global C1G2 DFSA identification algorithm for basic vehicles

**Fig 2:**
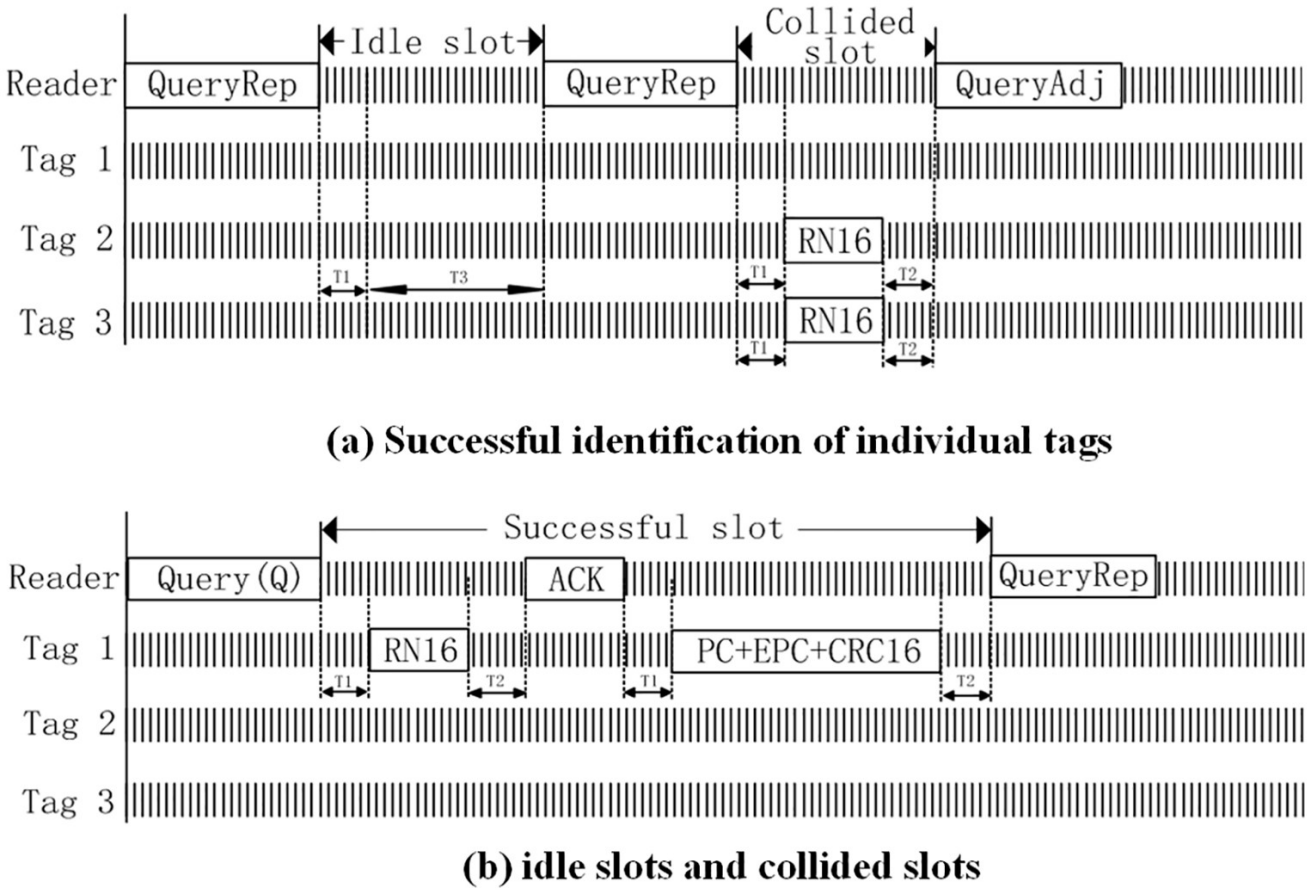
Possible results of a single slot in one frame. (a) Successful identification of individual tags. (b) idle slots and collided slots.

The execution process of the DFSA algorithm is to divide the identification time into several slots and specify several consecutive slots as one frame, with the value of frame length (L) as 2^*Q*^. where Q is the factor that decides the frame length when the reader communicates with the tag. In order to achieve the maximum efficiency of the system, the number of slots in the process of frame identification is adjusted by estimating the number of unidentified tags. Therefore, the frame length adjustment strategy and the tag number estimation algorithm are introduced into the algorithm proposed herein [40]. The specific execution process is as follows:

Step 1 The reader sends the Query command, mainly sending the value of Q(Q ∈ [0, 15], L = 2^*Q*^) to tags within the coverage of the reader.
Step 2 The tag will randomly select a number in the range of [0, 2^*Q*^ − 1] and store the selected number in the slot counter, and the tag which selects 0 will be the first one to respond to the reader.
Step 3 Later, the reader query on a given frame length slot by slot, and the tag with the number in the counter being 0 will responds to the reader by sending a 16-bit random number (RN16), which may produce three results:
  1. When there is no RN16 from any tag to respond to a time slot, it means that the slot is idle;
  2. When there are RN16 from more than one tag to respond to the slot, it indicates that slot collision occurs;
  3. When there is only one RN16 from one tag to respond to the slot, it means that the tag can communicate with the reader.
Step 4 After the reader confirms communication with the tag through the ACK command, the tag sends its own information to the reader, and the reader sends QueryRep command again to query the next slot. The detailed communication process is shown in Fig 2.

In the analysis of DFSA algorithm, assume that the frame length of the reader be L slots, and the number of tags identified by the reader in this range be N. As the probability that each slot is selected by a tag is 1/L, the probability of a single slot being selected by N tags at the same time is all the same. Therefore, the probability that a slot is selected by a known tag satisfies binomial distribution, and its expression is as follows:

$$P_{N,\frac{1}{L}}(n)=C_{N}^{n}{\left(\frac{1}{L}\right)}^{n}{\left(1-\frac{1}{L}\right)}^{N-n}$$ (3)

In a frame identification process, the identification probability *P*_*Succ*_ of a single tag can be expressed as (n = 1):

$$P_{Succ}=P_{N,\frac{1}{L}}(1)=C_{N}^{1}{\left(\frac{1}{L}\right)}^{1}{\left(1-\frac{1}{L}\right)}^{N-1}$$

or

$$P_{N,\frac{1}{L}}(1)=\frac{N}{L}{\left(1-\frac{1}{L}\right)}^{N-1}$$ (4)

Similarly, the occurrence probability *P*_*Idle*_ of idle time slot can be expressed as (n = 0):

$$P_{Idle}=P_{N,\frac{1}{L}}(0)=C_{N}^{0}{\left(\frac{1}{L}\right)}^{0}{\left(1-\frac{1}{L}\right)}^{N}$$

or

$$P_{N,\frac{1}{L}}(0)={\left(1-\frac{1}{L}\right)}^{N}$$ (5)

Therefore, the probability of slot collision can be expressed according to the occurrence probability of idle time slots and the successful identification probability of time slots:

$$P_{COLL}=1-P_{Succ}-P_{Idle}$$ (6)

In order to obtain the maximum throughput of the DFSA algorithm, the mathematical expectation of maximum throughput of the DFSA algorithm is obtained according to the probability equation of the current slot state. Based on this, the throughput of DFSA algorithm can be expressed as:

$$S_{Succ}=\frac{E_{Succ}}{L}=\frac{LP_{Succ}}{L}=\frac{N}{L}{\left(1-\frac{1}{L}\right)}^{N-1}$$ (7)

In Eq (7), *S*_*Succ*_ takes the derivative of L, and $\frac{dS}{dL}=0$, and the maximum value of *S*_*Succ*_ can be obtained when L and N satisfy the following relationship:

$$L^{2}(N-1)-(L-1)=0$$ (8)

The maximum throughput of the system when L = N can be obtained from Eq (8), and the maximum throughput is:

$$S_{max}=\frac{N}{L}{\left(1-\frac{1}{L}\right)}^{N-1}\approx e^{-1}\approx 0.3687$$ (9)

According to the above calculation process, we can know that the maximum throughput of the DFSA algorithm is 36.87% [41]. In order to maintain the maximum throughput of the DFSA algorithm, it is necessary to adjust the frame length L in real time according to the number of remaining tags after the execution of a frame identification algorithm. However, EPC Global stipulates the frame length L in the frame identification process should be 2^*Q*^, which cannot meet the requirement that L equals to N in real time. Therefore, the throughput achieved by the DFSA algorithm is generally lower than the theoretical value.

### Improvement of DFSA algorithm improving fast vehicle identification

According to analysis on execution efficiency of the DFSA algorithm in Section 2.1, there are two methods for improving the efficiency of DFSA algorithm: one is to keep the number of tags N basically consistent with the frame length L in the system execution process, so as to keep the maximum efficiency of the system unchanged, and it is difficult to improve the efficiency of the DFSA algorithm on the original basis; the other is to reduce collisions or idle slots in the identification process, but the slot collision occurs in DFSA algorithm is uncertain. As a result, this paper proposes a reservation-based DFSA algorithm to remove idle slots.

#### Principle of the reservation to cancel Idle DFSA algorithm

**Fig 3:**
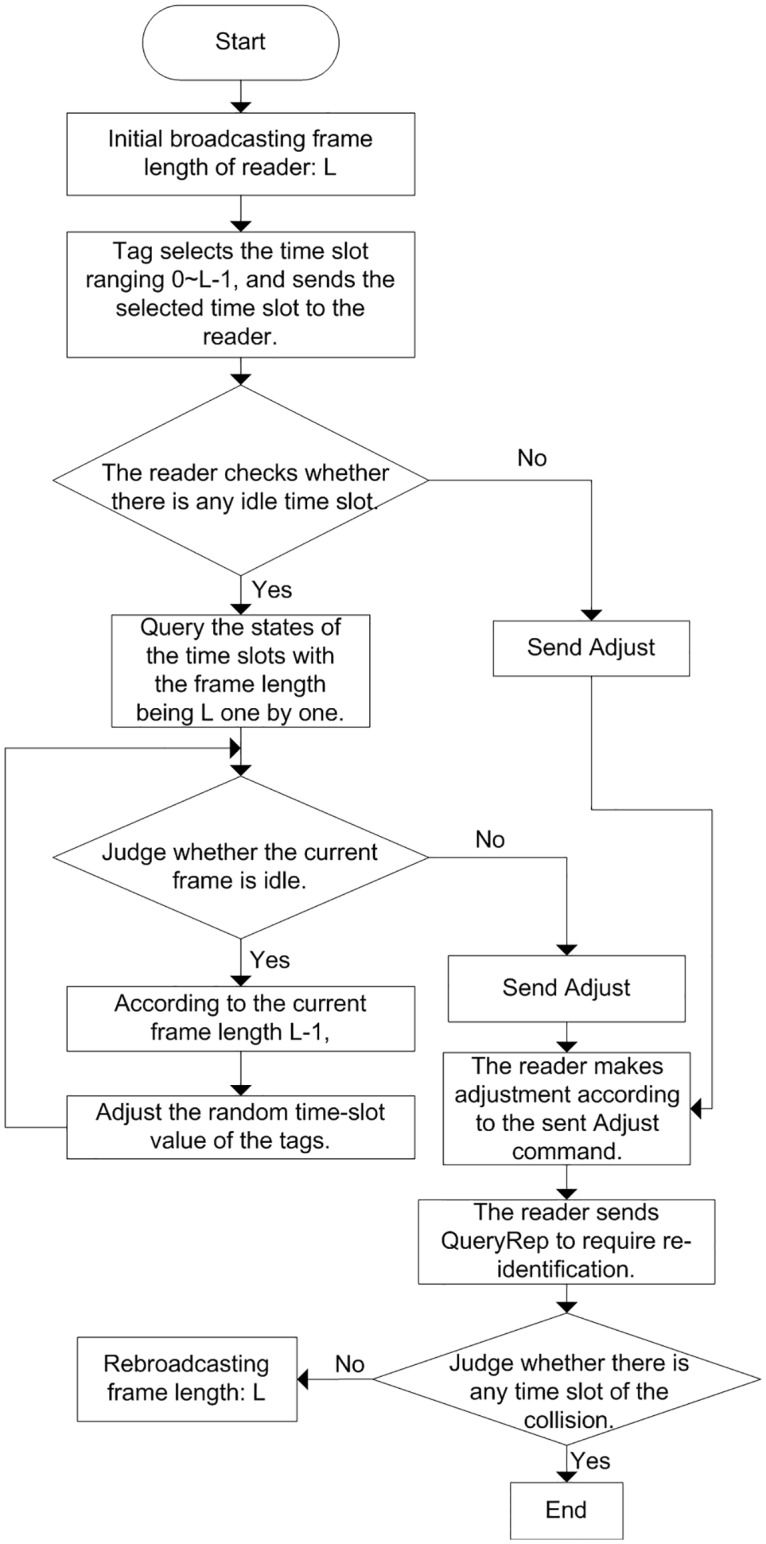
Reservation to cancel idle DFSA algorithm.

In order to improve the efficiency of the DFSA algorithm in the execution process, this paper proposes that the reservation idea is applied to remove idle slots in the frame. The reader reserve slots by broadcasting. Tags modify their randomly selected communication slots according to the predefined slots, thus shielding the idle slots in the frame and allocating only successful slots and collided slots. In this way, the improved DFSA algorithm is called the Reservation to Cancel Idle DFSA algorithm, and the specific steps as shown in Fig 3:

Step 1 The reader broadcasts an initial command Init (L), with L being the initial frame length.
Step 2 The tag in the identification area randomly select time slots ranging from 0 to L-1. If the tag chooses slot i to communicate with the reader, the corresponding slot value of the reservation sequence is set to 1 in the frame length of the reader, the value of the time slot reserved by the tag is stored in the counter, and the value in the tag counter is sent to the reader.
Step 3 The reader detects all the reservation sequences for receiving tags and queries the idle slots. If the current slot is found to be idle, the value of the corresponding slots in the frame is reduced by 1. After all the idle slots are queried, they are canceled in turn to obtain the final frame length for the execution process.
Step 4 The reader sends the reservation sequence of test results to the Adjust command of the tag, and the tag within the radiation range of the reader adjusts the internal count value according to the Adjust command. The adjustment process is as follows:
  1. The tag adjusts the value in the counter according to the frame slot returned by the reader, and maps the value in the original counter to get the adjusted value.
  2. After the tag adjustment are made according to the parameters in the Adjust command, a new slot sequence value is obtained, and the tag communicates with the reader in slot.
Step 5 The frame length *L*′ = *L*–*L*_0_ resent by the reader is the frame length after the idle time slot is canceled. The tag sends the corresponding frame length slot in the slot counter to EPC, and the reader completes the communication with all tags in one frame.

#### Improvement of the efficiency of the RTCI-DFSA algorithm

In the RTCI-DFSA algorithm, before the reader starts to identify the tag, it sends out an identification command with the frame length being L. The tag needs to randomly select the time slot ranging 0~L-1 as the communication time slot. Assuming the number of tags identified is N, the conclusion obtained in Section 2.2.1 shows that the probability of a single tag time slot to be identified is as follows:

P=1L (10)

In other words, the probability of a single slot to be selected is equal to the probability of successful tag identification:

$$P_{succ}=C_{N}^{1}P^{1}{\left(1-P\right)}^{N-1}$$ (11)

Therefore, the mathematical expectation *L*_*succ*_ of successfully identification of tags in a frame is:

$$L_{succ}=L\times P_{succ}$$ (12)

Similarly, the probability that a single slot is not identified can be expressed as follows:

$$P_{idle}=C_{N}^{0}P^{0}{\left(1-P\right)}^{N}$$ (13)

According to the above calculation results, the mathematical expectation *L*_0_ of the idle time slot in tag identification process is as follows:

$$L_{0}=L\times P_{idle}$$ (14)

When the reader detects an idle slot in the identification process, it only needs to send *L*_*all*_ = *L* − *L*_0_ slots for identification. So, the throughput of the system can be defined as the ratio of the successfully identified slots to the selected slots:

SRTCI-DFSA=LsuccLall+1=N×(1-1L)N-11+L×[1-(1-1L)N] (15)

Then, the identification rate of the RTCI-DFSA protocol is defined as the ratio of the number of successfully identified tags to the number of total tags N:

PRTCI-DFSA=L×SRTCI-DFSAN=L×(1-1L)N-11+L×[1-(1-1L)N] (16)

When the number of frame slots is equal to that of tags, the quantitative comparison between the efficiency of the RTCI-DFSA and that of the DFSA algorithm, that is, the equation for calculating system efficiency is as follows:

ML=N=L×(1-1L)N-11+L×[1-(1-1L)N]×LN×[1-(1-1L)N]=11L+[1-(1-1L)L] (17)

**Fig 4:**
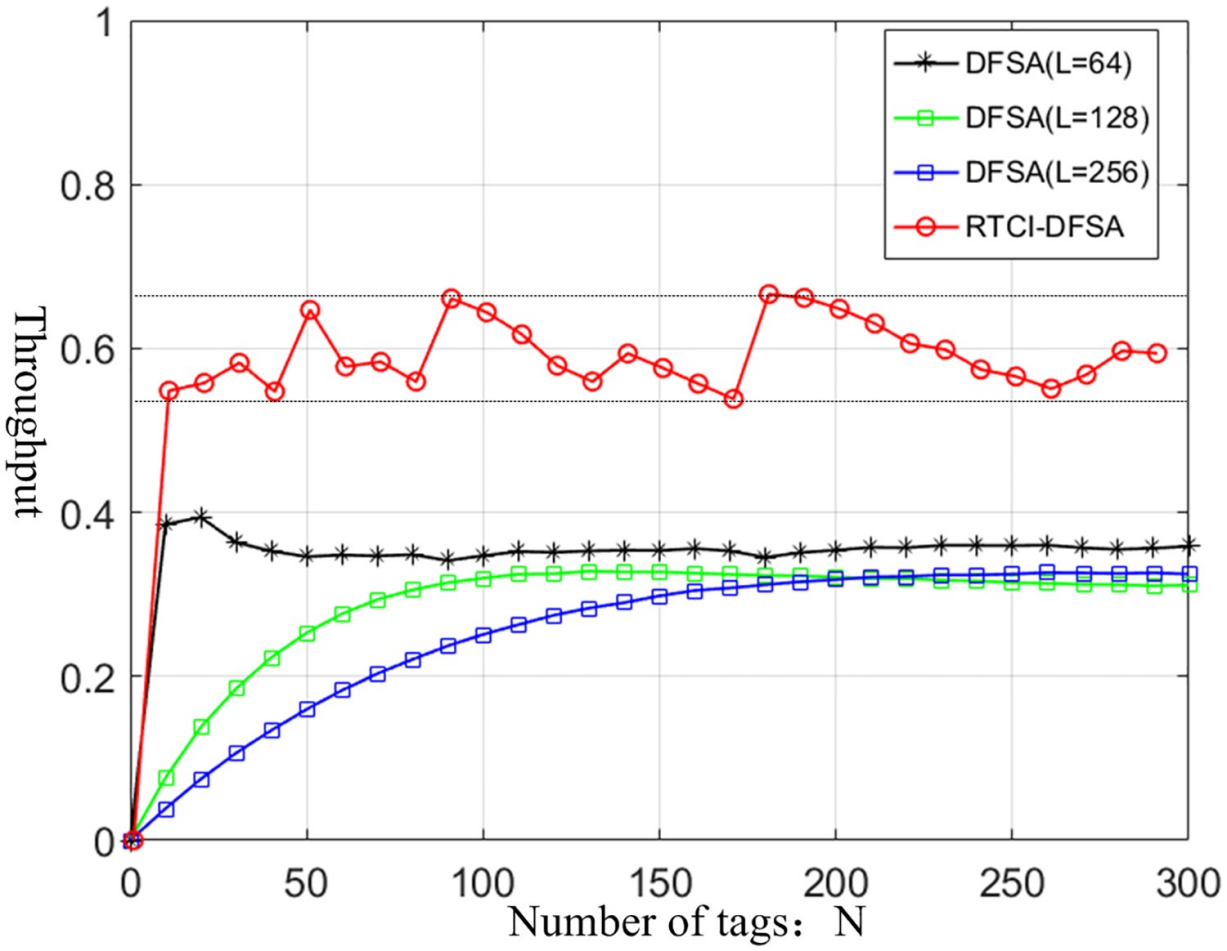
Identification efficiency of RTCI-DFSA system.

According to the above analysis, when L = N is determined, the simulation results of the RTCI-DFSA throughput are shown in Fig 4.

The simulation results of Fig 4 show that the identification throughput of the improved algorithm is obviously higher than 36.8%, and the system efficiency is related to the number of tags. When the Q value of the tag identification protocol is updated in the tag identification process, the system throughput is the highest and when the number of tags is equal to the length of the identified frame, the system efficiency is the highest.

## Construction of dynamic vehicle identification models

### Construction of dynamic RFID system model for vehicles

All previous tag anti-collision algorithms are applicable to a static RFID system, while the fast vehicle identification algorithm runs in a dynamic tag identification process. Though simpler than the dynamic RFID system, the existing static RFID system is not applicable to the dynamic tag identification process when tags are moving at high speed. Therefore, a dynamic tag identification model is introduced to address the fast identification of vehicles.

**Fig 5:**
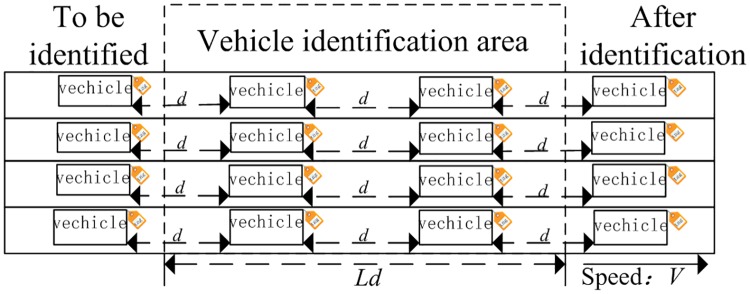
Basic vehicle identification model.

In the dynamic RFID system, it is assumed that firstly, vehicles continuously enter/leave the identification area according to a prescribed route with a constant speed; secondly, the number of vehicles passing through the identification area per unit time is uniform; thirdly, vehicles pass the reader identification range by following a prescribed direction towards the identification area; fourthly, each vehicle can only pass through the identification area once, and such RFID system is called a Dynamic RFID Identification System for Basic Vehicles. The reader identification range L_*d*_, vehicle density and speed within the identification range should be considered for the construction of a vehicle identification model within the reader identification range, and the specific model is shown in Fig 5.

The basic vehicle identification model in Fig 5 is abstracted and replaced by the basic conveyor model for analysis to facilitate the theoretical calculation in this paper. The rectangular boxes represent the vehicles running at a constant speed of V; *d* represents the distance between vehicles; Ld refers to the identification range of the reader, and the vehicles are equipped with RFID tags. Assume that after the system has been started for a while, the number of tags of the vehicles entering and leaving the identification area within a unit time does not change any more, and the identification rate of the system remains stable. Then, the system reaches a dynamic balance, and the reader identification range in the basic vehicle identification system is L_*d*_, the number of vehicles passing through the identification area within the unit time is *ρ*_*m*_, the vehicle speed is V, and the number of vehicles *M*_*v*_ identified by RFID within the reader identification range of the basic vehicle identification model is:

$$M_{v}=\rho_{m}\times L_{d}$$ (18)

Therefore, it can be derived from the model in Fig 4 that, the stay time of vehicles (t) within the reader identification area is:

$$t=\frac{L_{d}}{V}$$ (19)

### Efficiency of basic vehicle identification models

The RTCI-DFSA protocol, which improves the efficiency of systematic identification, is used for identification model of dynamic vehicle RFID system. However, the DFSA protocol is used for formula derivation in the calculation process to obtain determined results. According to the equation in the previous section, when the time slot size of system frames is *L*, and the number of unidentified tags is N, the throughput rate of the RFID system is:

$$S=\frac{N}{L}{\left(1-\frac{1}{L}\right)}^{N-1}$$ (20)

The number of tags specified in the identification process is indicated by *M*_*v*_, and the number of times of polling identification during the vehicle identification process is indicated by K. When the execution time of a single time slot of the basic vehicle identification system is *T*_*s*_, then the time (T) required for the identification of a frame by the tag is:

$$T=T_{s}\times L$$ (21)

It can be derived from the Eqs (13) and (15) that, the number of times of polling identification (K) on vehicles entering the reader identification range is:

$$K=\frac{t}{T}=\frac{L_{d}}{VLT_{s}}$$ (22)

According to the time required for the identification of each frame calculated during the identification process, it can be derived that the tag number (*m*′) of vehicles entering the reader identification area during one frame of identification process is:

$$m^{\prime }=V\rho_{m}LT_{s}$$ (23)

During the identification process, *m*′ of tags entering the reader identification range are taken as a tag identification group. The system identification rate of the tags is *P*_*DFSA*_, and the tags have undergone a total of K times of polling identification. Therefore, it can be derived that the tag identification probability P of a single vehicle is:

$$P=1-{\left(1-P_{DFSA}\right)}^{k}$$ (24)

The tags and tag groups within the identification range are always dynamically balanced when the system identification process reaches dynamic balance, then the identification rate of one tag group represents the identification rate of the system. Meanwhile, the throughput rate remains unchanged during the identification process of the system. Therefore, the number of identified/unidentified tags remains unchanged, then it can be derived that the number of unidentified tags *M*_*not*_ during the tag identification process is:

$$M_{not}=\sum_{i=1}^{k}m^{\prime }{\left(1-P_{DFSA}\right)}^{i-1}=\frac{m^{\prime }[1-{\left(1-P_{DFSA}\right)}^{k}]}{P_{DFSA}}$$ (25)

The final formula for the identification rate of RFID system can be obtained from the above Eqs (20), (22), (23) and (24):

$$P=\frac{N\times P_{DFSA}}{m^{\prime }}=\frac{S}{V{\rho_{m}T}_{s}}$$ (26)

According to the Eq (20), under the condition of dynamic balance, when the tag vehicle density is *ρ*_*m*_, the vehicle speed maintains at V, and unit slot length of the system maintains at *T*_*s*_, the tag identification probability in the vehicle basic identification model is proportional to the throughput rate of the system. It can be seen from the aforementioned analysis on the efficiency of DFSA algorithm that, the throughput rate of the system will reach a maximum limit when the number of the system identification tags (N) is equal to the frame length of the system (*L*). Therefore, the maximum efficiency of the basic vehicle identification model can be calculated as follows:

$$P_{max}\approx \frac{0.368}{V\rho_{m}T_{s}}$$ (27)

Therefore, according to the results of the Eq (27), it can be known that the maximum vehicle speed can be calculated when the system identification rate is 1. In order to ensure the vehicle identification efficiency, i.e. to ensure that there is enough time for the identification of vehicles within the identification range by readers, the vehicle speed should be:

$$V_{max}=\frac{0.368}{\rho_{m}T_{s}}$$ (28)

According to the analysis and calculation of the above equation, the identification efficiency of the system in dynamic balance is:

$$P=\{\begin{array}{ll} \frac{0.368}{V\rho_{m}T_{s}} & V>\frac{0.368}{\rho_{m}T_{s}}\\ 1 & V<\frac{0.368}{\rho_{m}T_{s}} \end{array}$$ (29)

Therefore, it can be seen from the Eq (29) that in the vehicle identification model of the system, the tag identification probability is only related to the tag vehicle speed V, vehicle density *ρ*_*m*_ with a specified range and time slot size *T*_*s*_. Next, we will introduce how to simulate the vehicle identification model designed in this paper, and determine the impact of each impact factor on the system identification efficiency through simulation.

## Collision prevention for fast identification of vehicles

### Loss of vehicle tags

In the previous section, vehicle identification is analyzed under the assumption that the vehicles have reached dynamic balance. This situation is more suitable for mathematical analysis in special circumstances, However, it is different from the vehicle identification model in practical applications, and it is difficult to directly find the identification efficiency of the system. Therefore, in order to solve the practical problem of vehicle identification, equal vehicle speed V, random number of vehicles entering the reader identification range and random entry time are adopted for the model calculation due to the fact that vehicle identification readers are installed in critical sections in highway, and that the vehicle speed is maintained within a certain range. Tag loss rate is also adapted to measure system performance to verify the practicability of the system [42]. In this section, the tag loss rate refers to the ratio of the number of unidentified tags to the total number of identification number in a group of vehicle tags. The tag loss rate is calculated by: subtracting the total number of identification tags from the number of identified tags, then dividing the total number of identification tags by the calculated value. It can be seen from the previous calculation that the system tag identification is proportional to the throughput rate.

**Fig 6:**
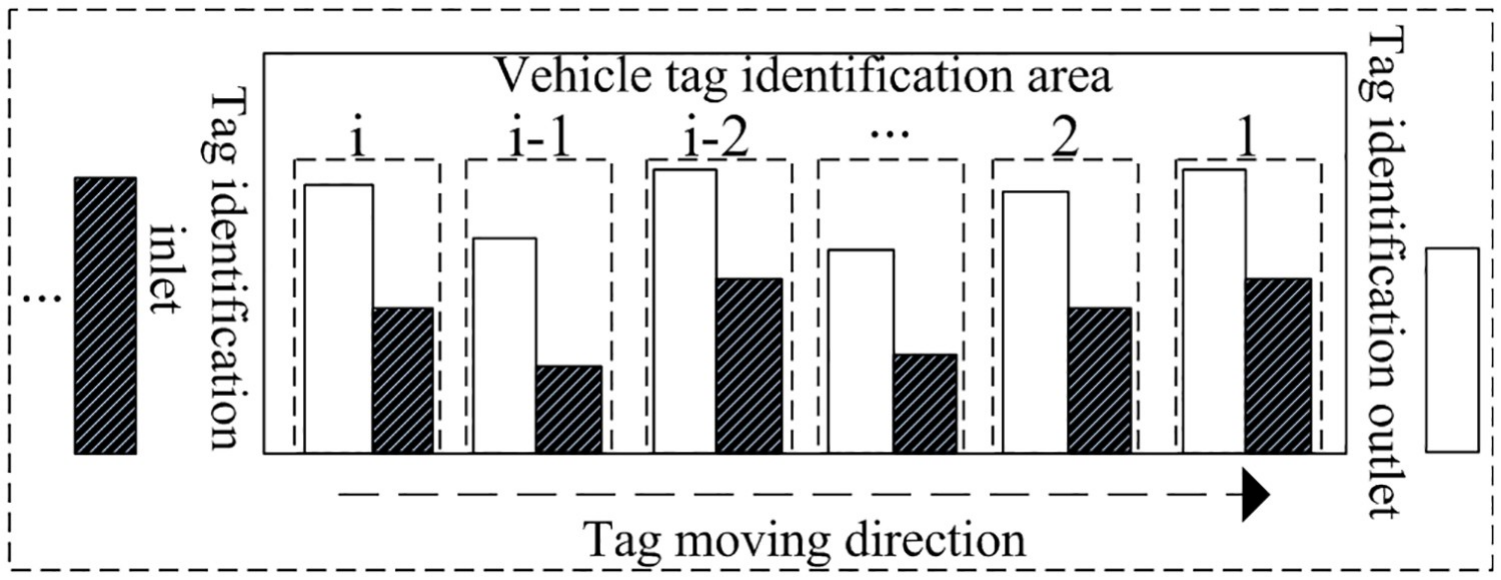
System tag group identification model.

In Fig 6, the rectangular frame area indicates the tag group i of vehicles entering the identification area, the white area indicates the number of identified vehicle tags, the black area indicates the number of unidentified vehicle tags, which is proportional to the number of tags of the whole group, and the horizontal ordinate indicates the time the vehicle entering the identification area. Different from the basic vehicle identification model mentioned in the preceding section, the vehicle identification system takes into account the multi-tag grouping of vehicle tags, and the number of tags is random, variable and distinguishable, which distinguishes the uniform tag density and same frequency of tags entering the identification area in the basic vehicle identification. Therefore, new problems, such as identification of tag groups, random quantity of tags and interval of random slots are generated in the rapid vehicle identification system, which leads to the disorder of tag identification sequence and tag loss or other problems during the system identification process. A lost tag refers to a tag that leaves the identification area without being identified after it enters the identification area.

### Disorder of random identification sequence of vehicle tags

**Fig 7:**
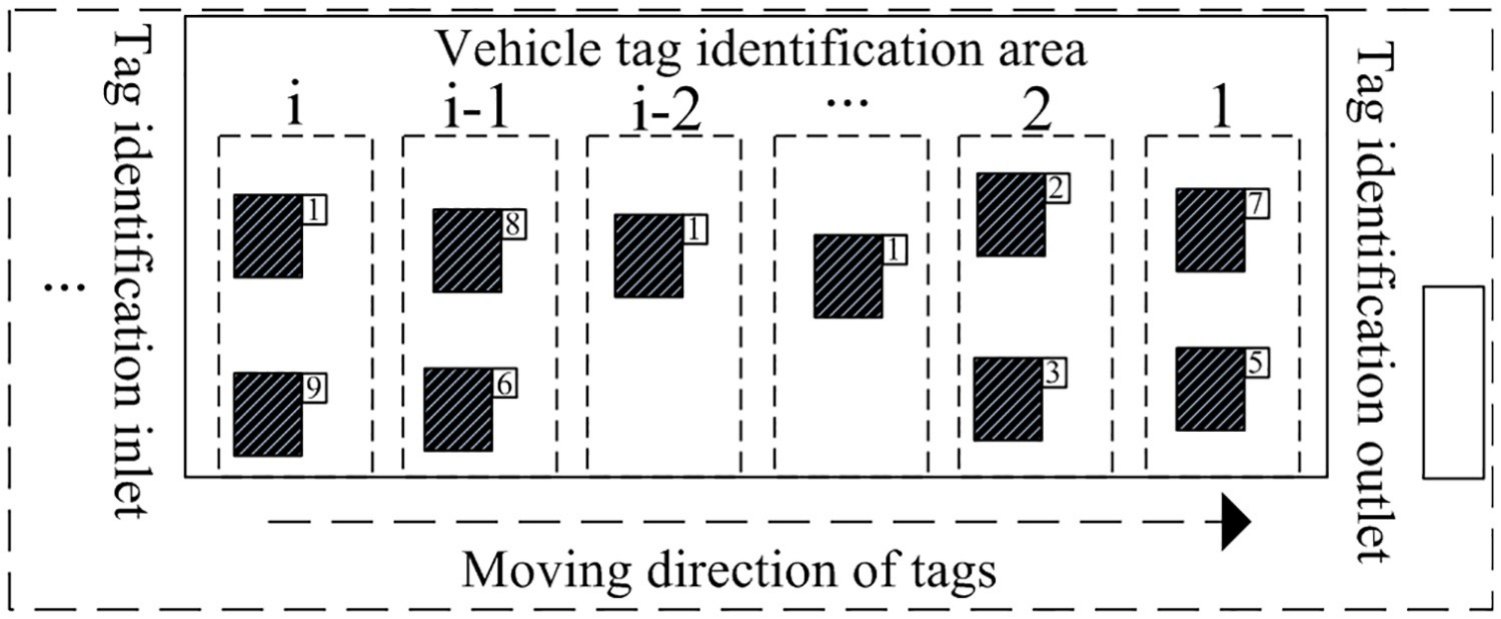
Selection of time slot for vehicle tag identification.

Disorder of random identification sequence is the most typical problem of IVIS, and it is an important determinant of vehicle tag identification protocol in the system. Therefore, this problem should be solved first after the addressing of the efficiency of tag collision. Descriptions of the identification system model is as follows:

It can be seen from Fig 7 that when the reader polls for one circle, the tag group in the identification area will select a slot number. In the figure, the slot numbers 7 and 5 were selected by the tag group 1, which firstly entered the identification range and should leave first. However, during the execution of identification algorithms, the reader will first process the tag groups with smaller time slot number. Therefore, the tag that first enters the identification area (like the tag group 1 in this example) may leave the reader identification range before being detected during the polling, and become a lost tag. This phenomenon is caused by the disorder of random identification sequence resulting from the randomness of the vehicle tags and the validity of the stay time.

Therefore, the loss rate of vehicles identification system can be reduced by increasing the stay time of tags in the identification range and weakening the randomness of tags. However, speed of vehicle tags in the identification range and the radiation range of readers are determined by the vehicle speed and the system itself, which are difficult to control, so the method of weakening the randomness of tags to change the loss rate of system tags is adopted in this paper.

### Tag serialization incorporating reservation to cancel idle

In order to eliminate the impact of the value of randomly obtained slot on the tag polling times when the tags enter into the reader identification range, the tag groups of moving vehicles are serialized, and tags are processed in the principle of “first come, first out” based on the serialization of tag groups. Tags entering at different time can be grouped into one group, that is, assign the new tags with same time sequences every a fixed time period. Tags entering the system in the same period will be assigned to the same sequence. Thus, even if the number of tags entering into the reader range is different at every equal time interval, the tags entering the system at the same period will have the same time sequence, which facilitates the sequence of tags during the system identification process.

**Fig 8:**
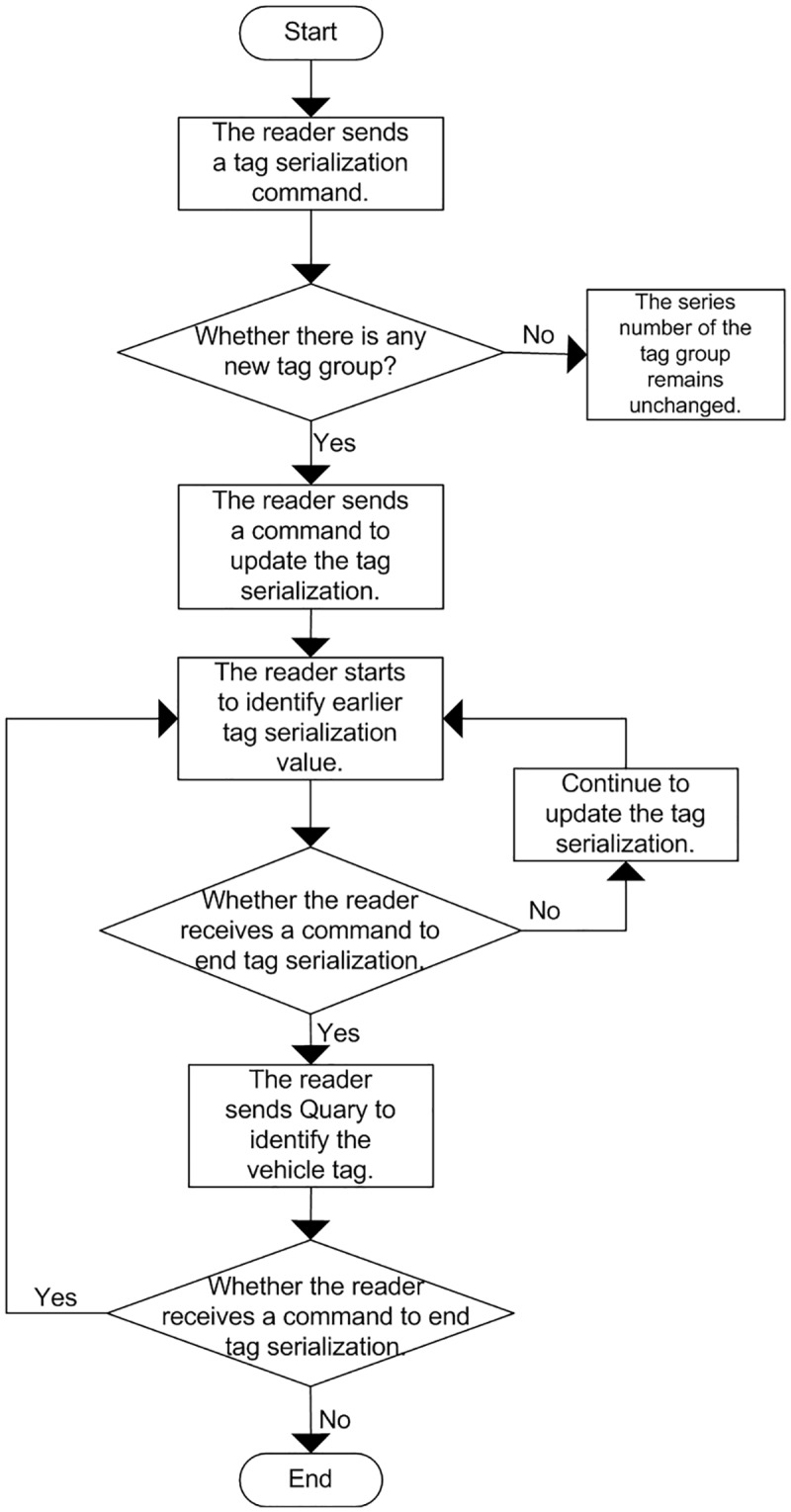
Serialization process incorporating reservation to cancel idle for tag identification.

When introduced, the tag serialization method incorporates the reservation to reduce the tag loss rate of the system, and the detailed tag identification process is as shown in Fig 8.

The detailed steps of serialization process incorporating RTCI-DFSA for tag identification in Fig 8 are as follows:

Step 1 A tag serialization command is sent when the reader starts, where the initial identification sequence is 0, and there is no tag sequence entering into the reader identification range.
Step 2 It is checked whether the updated “Head” value sent by the reader is the same as the serialized tag value “Round” in the identification range. If it is the same, it indicates that there is no new tag sequence enters into the identification range, then perform Step 1 in sequence.
Step 3 The tag group that first enters the reader identification range is identified. The parameter Head command is used to match the earliest entered tag group. If the tag group is lost, then proceed to the next cycle to identify the tag group.
Step 4 A Query command will be sent by the reader to identify the tag arrays within the effective identification range, and the frame length of the parameter L is set to 2^*Q*^.
Step 5 The range of slots selected by the sequence group with the smallest tag within the reader identification range is 0—L. According to the sequence value reserved by tags, the time slot position is “Position 1”, then the number of time slots is stored in a register during the reservation, and the Position 1 will communicate with the tags.
Step 6 After the reader has received all the tag sequences, it will execute the command of removing idle slots to update the effective frame length in the reader identification.
Step 7 The reader will re-determine the number of remaining tags in the identification range and re-update the tag sequence as the initial identification value for the next polling identification.

## Simulation analysis of intelligent vehicle identification system for expressways

### Simulation of basic vehicle identification efficiency under dynamic balance

In this paper, it is necessary to carry out simulation on the tag loss rate in the basic vehicle identification model and improved algorithms to verify the identification rate of tags in the dynamic vehicle identification system.

The computer used in the experiment, with a central processing unit of Intel i5-6500 and a memory of 4GB, is equipped with a win7 operating system, to carry out simulation under the operating environment of MATLAB.

**Fig 9:**
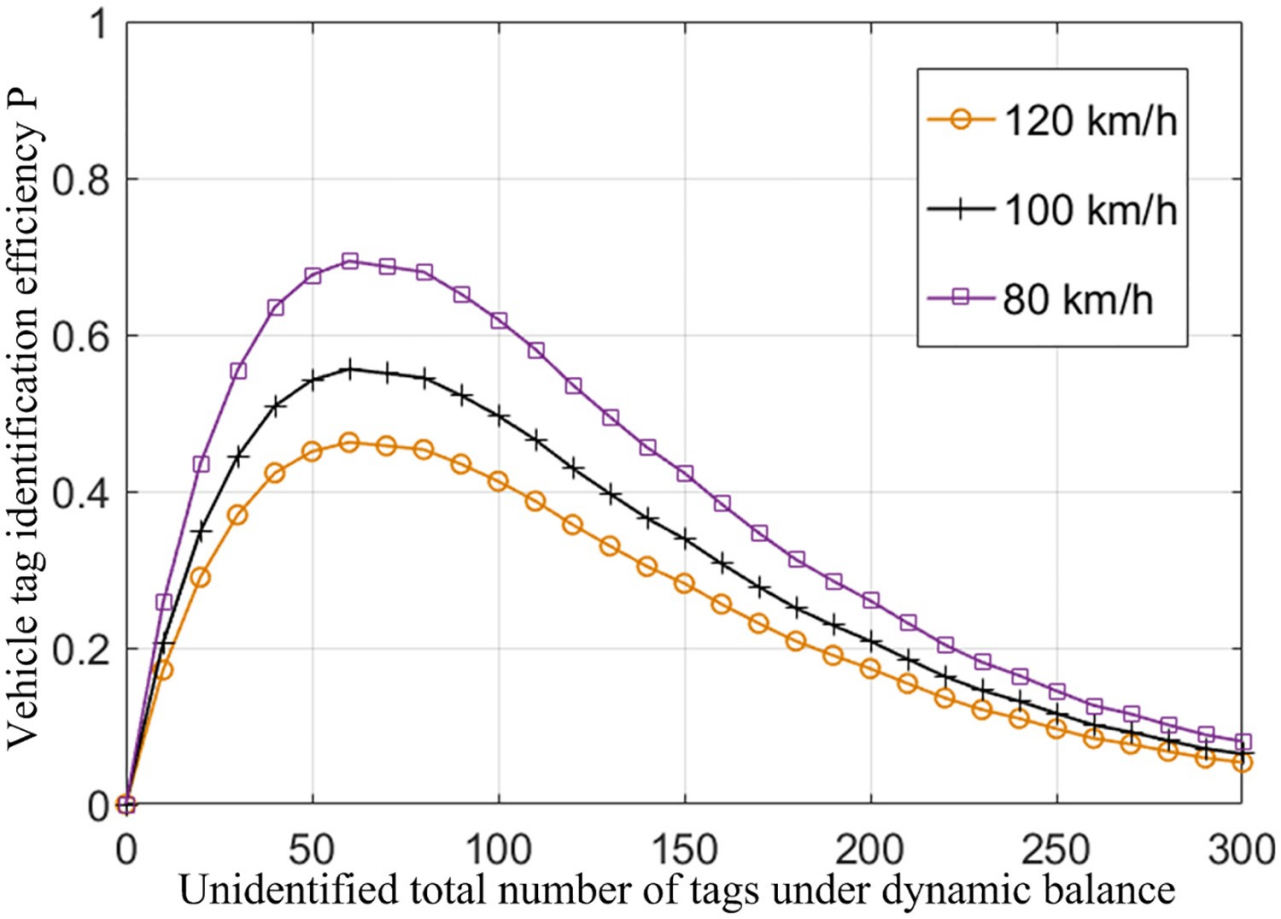
Identification rate of vehicles at different speeds under dynamic balance.

It can be seen from the previous theoretical analysis on the basic vehicle identification model that, if the vehicle tag density is *ρ*_*m*_, the vehicle speed is V and the frame length L is constant, then the basic vehicle identification model can achieve dynamic balance, and calculate tag identification rate with the V, *ρ*_*m*_, L and *T*_*s*_ unchanged. In order to carry out simulation under the state of dynamic balance, the following conditions are assumed:

1. The tag density of running vehicles is 12 /m and remains unchanged;
2. The length of the reader identification area *L*_*d*_ is 20m, that is, the effective length of the tag identification;
3. In order to obtain a specific simulation result, the FSA algorithm is used to identify tags in the identification area, the frame length of tags identified by a reader is 64, and the size of each time slot *T*_*s*_ is 0.001s.
4. The simulation should be carried out separately at different vehicle speeds while meeting the above conditions, and the results of simulation on system efficiency at different vehicle speeds are shown in Fig 9.

It can be calculated from the number of times of tag polling k=LdVLTs≈312V within the reader identification range that, in the identification system, the number of times of tag polling each time the tag group enters the identification zone to leave the identification zone is related to the speed of the vehicle. Therefore, according to the latest speed limits for different sections of expressways in China, if the design speed is 80 km/h, the maximum speed limit is raised to 100 km/h; if the design speed is 100 km/h, the maximum speed limit is raised to 120 km/h; and the tunnel speed limit is raised to 80 km/h. So, in our experiment, three speed is selected for discussion.

As shown in Fig 9, the identification efficiency of the system increases as tag movement speed decreases. In the identification process of high-speed vehicles, when the speed V is the only variable factor of the system, the faster the vehicle tags move, the lower the identification rate of the vehicles will be.

The efficiency of the system calculated by the RTCI-DFSA algorithm when the system reaches dynamic balance is compared in the simulation analysis of different protocols in the RFID tag mobile identification system under dynamic balance, and it can be seen from the previous Eqs (24) and (25) that, the identification rate of tag groups in the basic vehicle model is:

$$P_{RTCI-DFSA}=1-{\left(1-P_{RTCI-DFSA}\right)}^{k}$$ (30)

Similarly, it can be calculated from the Eq (25) that, the total number of unidentified tags *m*_RTCI−DFSA_ within the identification range is:

$$M_{RTCI-DFSA}=\frac{m^{\prime }[1-{\left(1-P_{RTCI-DFSA}\right)}^{k}]}{P_{RTCI-DFSA}}$$ (31)

Similarly, it can be calculated from the equations that, the system identification efficiency P_RTCI−DFSA_ of RTCI−DFSA in the basic vehicle identification model is:

$$P_{RTCI-DFSA}=\frac{M_{RTCI-DFSA}P_{RTCI-DFSA}}{V{\rho_{m}LT}_{s}}=\frac{S_{RTCI-DFSA}}{V{\rho_{m}T}_{s}}$$ (32)

**Fig 10:**
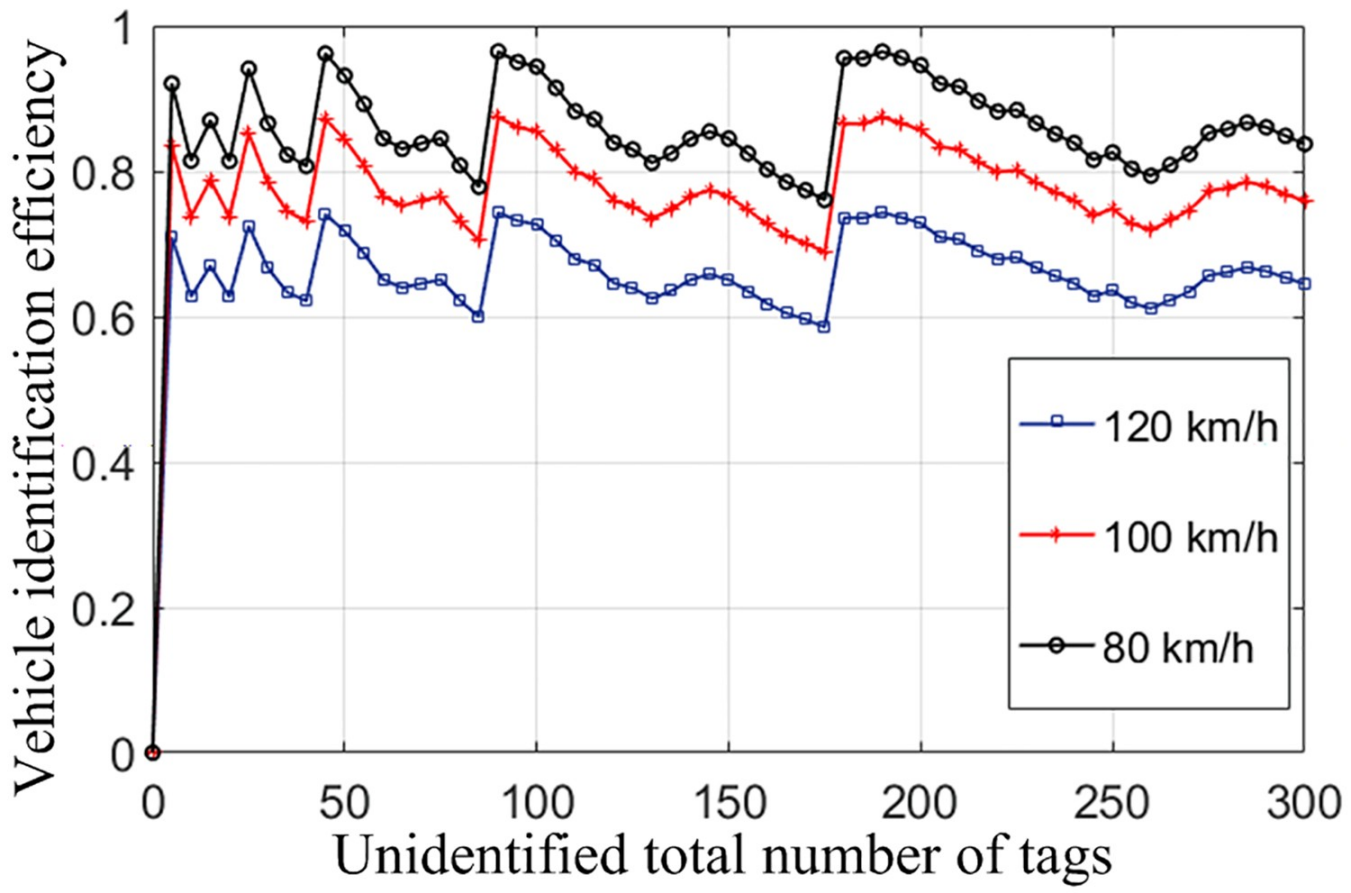
Identification rate of vehicles at different speeds under the RTCI-DFSA algorithm.

Therefore, according to the previous assumptions, the system efficiency at vehicle speeds of 120km/h, 100km/h or 80km/h during the simulation design process is:

It can be seen from the identification efficiency of the system in Fig 10 that, when a vehicle runs at a low speed, the vehicle identification rate can be up to 0.8595, and when the vehicle runs at the top speed of 120Km/h, the identification rate will be as low as 0.5565. The system identification rate is significantly higher than that of the pure DFSA algorithm. The improved protocol improves the throughput rate of the system. When the vehicle tags are identified, the number of times of tag polling within the identification range will increase due to the absence of time slots, which greatly improves the execution efficiency of the algorithm.

### Simulation for fast identification of vehicles

**Fig 11:**
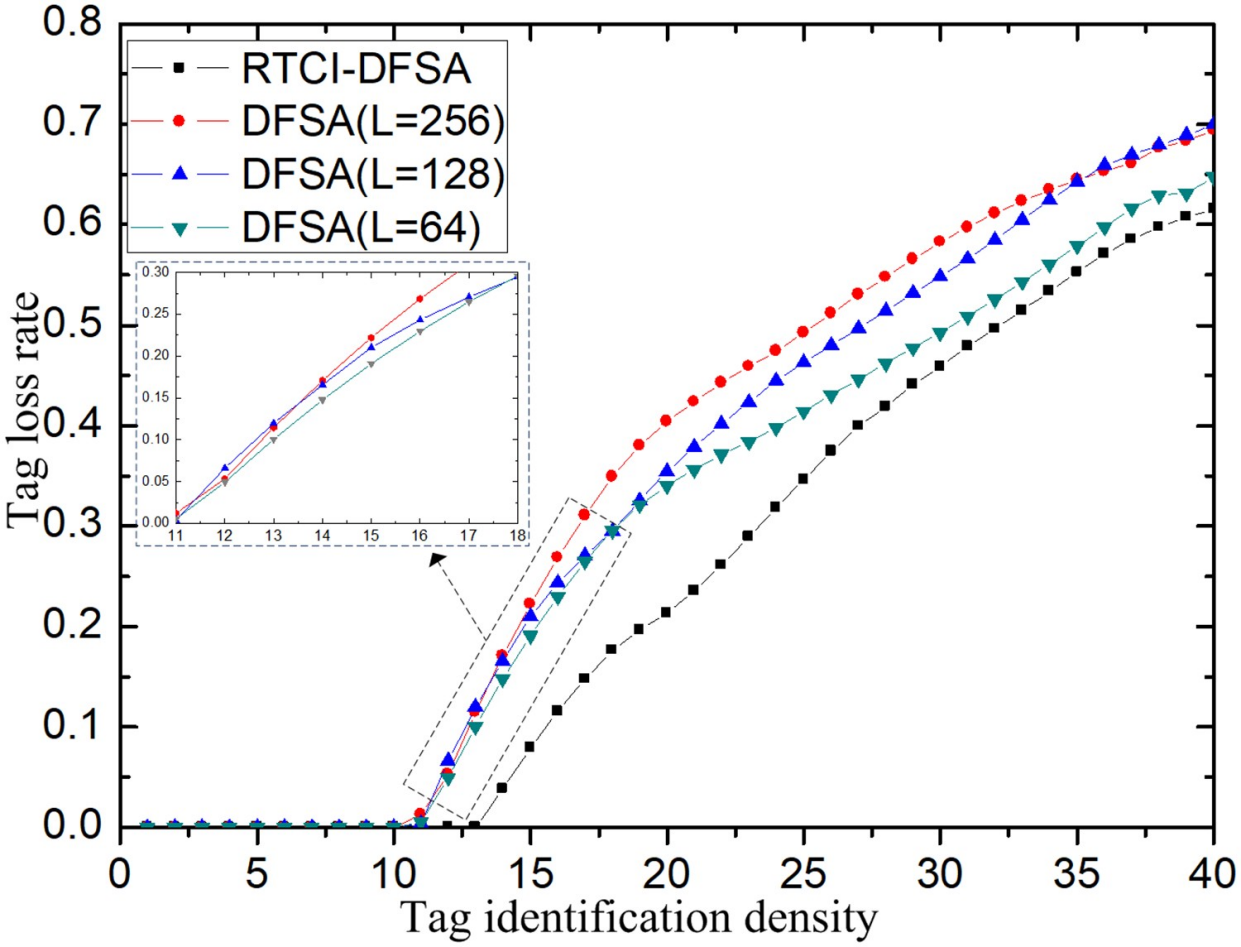
Relationship between tag density and tag loss rate.

In the calculation, tag loss rate can only be realized through the dynamic Markov chain calculation under FSA fixed frame length protocol. In this case, a huge calculation volume is involved, and it is difficult to obtain an effective tag loss rate. In order to verify the advantages and disadvantages of the tag serialization model incorporating the reservation to remove idle slot, the experimental simulation was adopted to calculate the tag loss rates and the tag loss rates of the system under different protocols were compared in this paper. The simulation model was simplified to obtain the simulation results, with detailed description of the simplified simulation model as follows:

1. In the identification model of simplified vehicle, the loss rate of vehicle tags was simulated for different vehicle tag densities;
2. T_slot represents the time slot of tag identification. T_slot can reflect the stay time and passing speed of tags in the identification range of the reader, and can be used to get the polling times of tags in the range of the reader *ρ*_*m*_;

The simplified model adopted in the simulation process considered only the time when the tag sends information to the reader, instead of the time when the reader sends commands to the tag. In order to verify the validity of the results, the frame length L of the system in the experiments was set as 64, 128 and 256, T = 0.01s, and the passing speed of vehicles is set at 120km/h. Other parameters are all fixed values, and the simulation results are obtained through qualitative analysis to reflect the actual effectiveness of the system. Other values may also be selected to simulate in the process. However, we compare the advantages of the improved algorithm on the condition that other parameters remain unchanged. The simulation results are shown in Fig 11.

Fig 11 shows that the tag loss rate is related to the tag density. The higher the tag density, the higher the tag loss rate. When the tag density is higher than 10, the tag loss rate is 0. By comparing the loss rates under several protocols, it is concluded that the tag loss rate of the improved RTCI-DFSA algorithm proposed is much lower.

**Fig 12:**
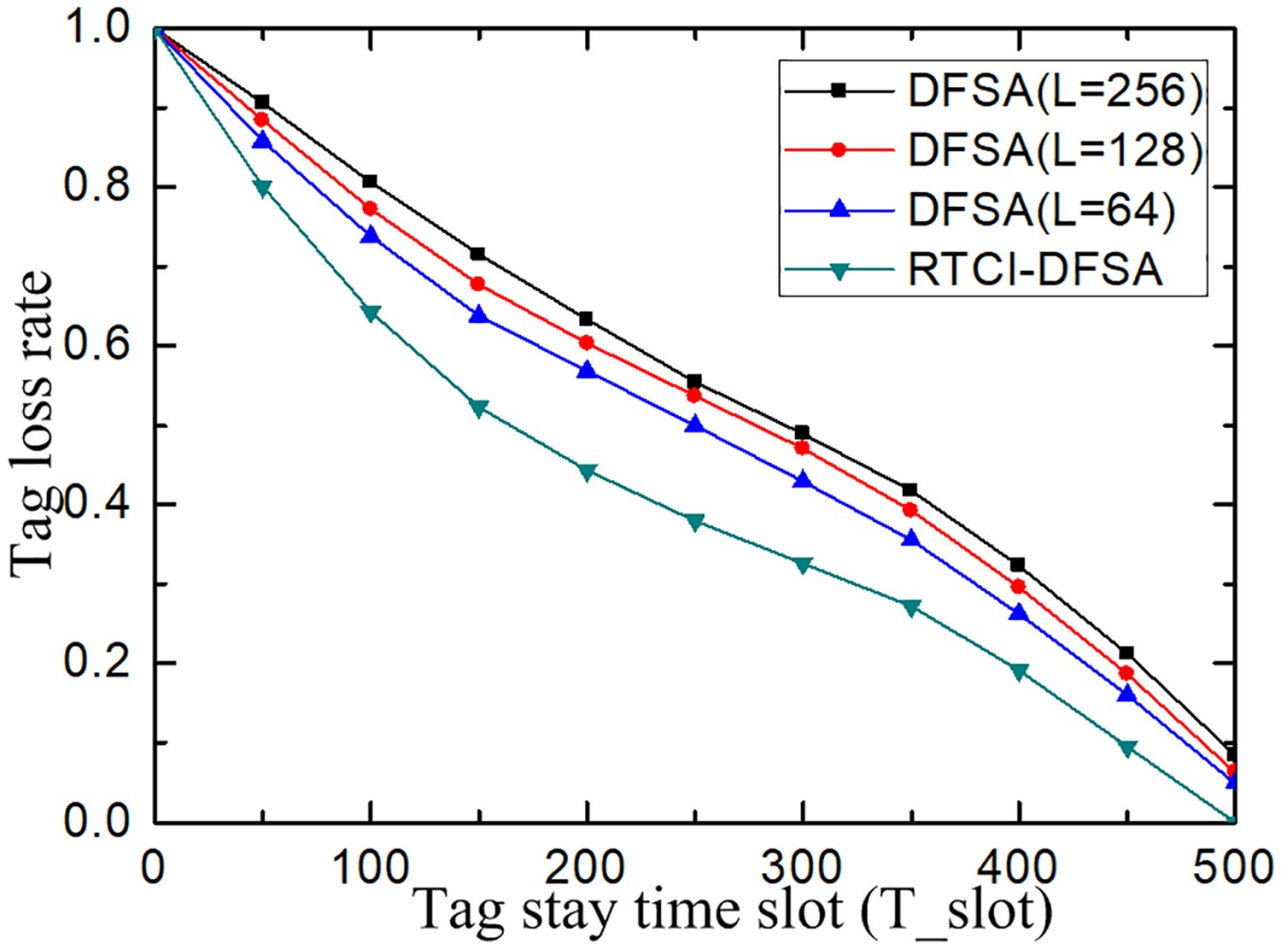
Relationship between tag stay time slot and tag loss rate.

Similarly, in order to verify how the stay time of different tag groups in the identification range of the reader influences the tag identification rate of the system, the slot value T_slot was changed for simulation. The larger the value T_slot, the longer the stay time of the tag groups in the identification range, and the lower the frequency of tag groups entering the identification range. The applicability of the improved algorithm to the fast vehicle identification is verified by simulation experiments, and the simulation results are shown in Fig 12.

Fig 12 indicates that the stay time of tags is related to their speed. From the comparison of simulation results, it can be seen that the tag loss rate decreases with the increase of stay time of tags in the identification range as the polling times of tags increases in the identification range. The simulation results under the tag identification protocol incorporating the reservation to reservation to cancel idle slot are significantly lower than that of the DFSA algorithms. This is because that the advanced tag identification mechanism adopted by the tag serialization helps to improve the tag identification efficiency and is applicable to the fast vehicle identification system adopted by the expressway.

## Conclusion

Fast and accurate vehicle detection on expressways in harsh environment is critical for ensuring roadway safety. We have proposed a novel RFID-based IVIS framework to get rid of strong interference and tag collision on freeways. More specifically, a modified DFSA-based algorithm (RTCI-DFSA) has been designed to identify vehicles in highways. Secondly, a fusion tag serialization identification method has been developed to reduce the loss rate of vehicle tags, and an advanced identification mechanism was introduced to further enhance the performance. Finally, in order to verify the applicability of the method, the recognition rate of the system measured under different vehicle speeds is increased to 0.6. The loss rate of different vehicle label density simulation systems is applied, and the vehicle tag density is increased to further challenge the model performance. We observed that when the tag loss rate reached zero which effectively validated the efficiency of the expressway IVIS. In sum, the proposed framework achieved good results in both theoretical analysis and simulation aspects, and can meet the requirements of rapid identification of vehicles in expressways. In future, we can expand our research by introducing deep learning based methods to further enhance the model robustness under extreme weather conditions and strong electromagnetic interference.

## Supporting information

S1 Dataset Original data sources. (RAR)
